# Trips for Health and Pleasure

**Published:** 1906-10-13

**Authors:** 


### TRIPS FOR HEALTH AND PLEASURE.

### THE ISLAND OF CAPRI.

When writing on the subject of Capri, it is impossible not to turn to that admirable work, "The Book of Capri," by Harold Trower, an author who is British Consul on the Island and whose long residence there enables him to speak with confidence on everything connected with it. The Island rises, as Mr. Trower puts it, "like an Alpine rock at the extreme south of the Gulf of Naples"; and it is about twenty miles from that fine southern city—the largest in Italy.

The island has long been celebrated as a health resort, and justly so. There is not a patch of marshy ground on it, and anything in the shape of epidemic disease is practically unknown. There is a freedom from dust, the climate is mild, and there is much sunshine. Not that the climate is perfection during the winter and spring months, but it is pointed out that Capri "is small enough to feel the full effects of the sea in producing equability of temperature throughout the year." That is one of its immense advantages to the invalid; and another, and not less important one, is that the diurnal range of temperature is small. Furthermore, the barometer remains fairly steady, being about 30 inches for such opposite months as January and June, and the slight variations from that reading point to the prevalance "of anti-cyclonic conditions or fine weather." Still there is a fair amount of rain, and at times a good deal of wind. Fortunately the lime-stone formation allows the rain to run off the surface and the inconvenience of muddy roads is not felt. As a rule it may be said that the temperature stands about 9° higher than in London. Consumptives would not find it suitable, except, perhaps, in the very earliest stage. There are several medical men in Capri, all of whom speak English, and one is an Englishman.

To the historian, the antiquary, the artist, the botanist, the yachtsman, and, in a mild degree, the sportsman, the island is full of interest. The invalid need not feel bored for want of something to interest him. During the winter months when hotels and pensions and villas are full of visitors and the studios in full swing, there is plenty of society, and although it may occasionally verge on the Bohemian variety it is not less amusing on that account.

Mr. Trower devotes a chapter of his book to the famous Blue Grotto, which he tells us is visited annually by 35,000 people, and as each visitor has to pay a franc and a half, the little island draws an income of £1,750 a year from this source. The water in the grotto does certainly appear of a wonderful blue colour; bluer even than the little lake near Kandersteg. Another chapter gives a short and very clear description of the Mithraic Cave on the south side of the island; and as the traveller explores that cave and afterwards examines the bas-relief taken from it and now in the Naples Museum, he may forget for a minute that he is a twentieth century tourist, and may ponder over the time when that extraordinary Mithraic religion found its way across western Europe, even to the shores of Britain.

There are many routes by which the invalid may reach Capri. If a good sailor he will take an Orient liner from London to Naples, or a North German-Lloyd one from Southampton at a cost of about £14 or £15; while he who dislikes the sea will go overland to Naples. The first route will take about eight days, and the latter means about fifty consecutive hours of railway travel unless a break be made somewhere on the way, say, at Milan, Genoa, or Spezia.

A compromise may be effected by going overland to Marseilles, and then taking one of the English or French steamers which will land him at the Immacolatella pier in Naples in thirty-six hours, to which he must add the twenty-four hours from London to Marseilles. These journeys are all rather expensive, but when once Capri is reached living is not expensive—is, in fact, cheaper than in most health resorts. The best hotels are the Quisisana (120 beds), Pagano's Hotel, Hôtel Bristol; Hôtel Londra, and the Hôtel Paradiso. There are also pensions and cafés.

Capri is a most convenient centre for excursions. Naples is only two hours' sail, and Rome five hours by rail from Naples. There is a good service of steamers from Naples to almost every seaport in Italy and Sicily, and to Malta, Alexandria, Smyrna, Constantinope, etc.

We strongly advise the invalid who intends wintering in Capri to provide himself with a copy of Mr. Trower's book. It is a work which may be read through and referred to again and again; and it is so nicely illustrated and so well bound that it is quite worthy of a place in any library. It is not an ordinary guide book.

